# Tinker, tailor, soldier, cell: the role of C-type lectins in the defense and promotion of disease

**DOI:** 10.1093/procel/pwac012

**Published:** 2022-07-15

**Authors:** James N Arnold, Daniel A Mitchell

**Affiliations:** School of Cancer and Pharmaceutical Sciences, King’s College London, London SE1 1UL, UK; Warwick Medical School, University of Warwick, Coventry CV2 2DX, UK

**Keywords:** C-type lectins, DC-SIGN, MBL, selectins, infection, arthritis, cancer

## Abstract

C-type lectins (CTLs) represent a large family of soluble and membrane-bound proteins which bind calcium dependently via carbohydrate recognition domains (CRDs) to glycan residues presented on the surface of a variety of pathogens. The deconvolution of a cell’s glycan code by CTLs underpins several important physiological processes in mammals such as pathogen neutralization and opsonization, leukocyte trafficking, and the inflammatory response. However, as our knowledge of CTLs has developed it has become apparent that the role of this innate immune family of proteins can be double-edged, where some pathogens have developed approaches to subvert and exploit CTL interactions to promote infection and sustain the pathological state. Equally, CTL interactions with host glycoproteins can contribute to inflammatory diseases such as arthritis and cancer whereby, in certain contexts, they exacerbate inflammation and drive malignant progression. This review discusses the ‘dual agent’ roles of some of the major mammalian CTLs in both resolving and promoting infection, inflammation and inflammatory disease and highlights opportunities and emerging approaches for their therapeutic modulation.

## Introduction

The relationship and interaction between the mammalian host and infectious microorganisms is deep and dynamic, involving hundreds of sensing molecules, signaling cascades and physiological responses. Mammals have evolved alongside infectious pathogens which has shaped their immune system and responses to infection. However, in doing so, pathogens have also developed sophisticated methods to avoid or exploit these defenses to facilitate infection and help circumvent elimination.

“*We*’*ve spent our lives looking for the weaknesses in one another*’*s systems*” Tinker, Tailor, Soldier, Spy by John Le Carré (1974) .

C-type lectins (CTLs) are a superfamily of proteins which contain one or more CTL-domain (CTLD) (Drickamer, 1999; Brown et al., 2018). The CTL family consists of over 1000 proteins split into 17 subgroups and are referred to broadly as “C-type” for the calcium dependency of their glycan interactions (Weis et al., 1992; Cummings and McEver, 2009). However, for some CTLs their CTLD can also bind non-sugar moieties (Cummings and McEver, 2009).

**Figure 1.**
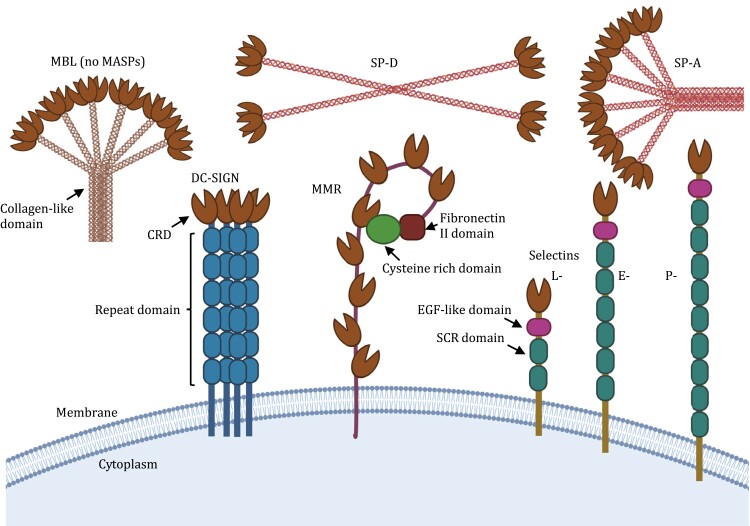
**CTL structures**. A cartoon representation of the domain structure of CTLs and CTLRs discussed in this review. The collagen like domains depict the triple coiled helix of three individual subunits that then associate to form higher order oligomers. CRD, carbohydrate recognition domain; DC-SIGN, dendritic cell-specific intercellular adhesion molecule-3-grabbing non-integrin; EGF, epidermal growth factor; SCR, short consensus repeat domains; MBL, mannose-binding lectin; MASPs, MBL-associated serine proteases; MMR, macrophage mannose receptor; SP-A/D, surfactant protein A/D. The image is not drawn to scale. Created using BioRender.com.

In mammals, CTLs can be both secreted soluble proteins or transmembrane cell surface receptors (Fig. 1) and have calcium-dependent lectin binding specificity for mannose (Man), *N*-acetylglucosamine (GlcNAc), and fucose (Fuc) (Weis et al., 1992; Mitchell et al., 2001; Arnold et al., 2006a). CTLs play significant roles in immunity including both humoral and cellular compartments and provide an innate first-line defense against pathogens. However, as our understanding of CTLs has developed it has become apparent that their roles extend beyond simply identifying and helping to fight infection. In this review, we consider the double-sided role of CTL interactions in the identification of both pathogen- and host-associated glycoproteins (Malhotra et al., 1995; Arnold et al., 2006a), but also in providing both protection and facilitation of infection and inflammatory disease (Geijtenbeek et al., 2000b; Santos et al., 2001). This review considers how CTLs can act as both “friend” or “foe” in certain biological contexts, focusing on the most well-characterized CTLs including mannose-binding lectin (MBL) (Sim et al., 2016) and CTL receptor (CTLR) dendritic cell-specific ICAM-3-grabbing nonintegrin (CD209; DC-SIGN) (Mitchell et al., 2001) (Fig. 1). A greater understanding of the CTL protein family and their dual-sided “spy networks” could help to identify therapeutic checkpoints, wherein CTL function could be modulated to drive beneficial outcomes.

## DC-SIGN in host dynamics and infectious disease

**Figure 2.**
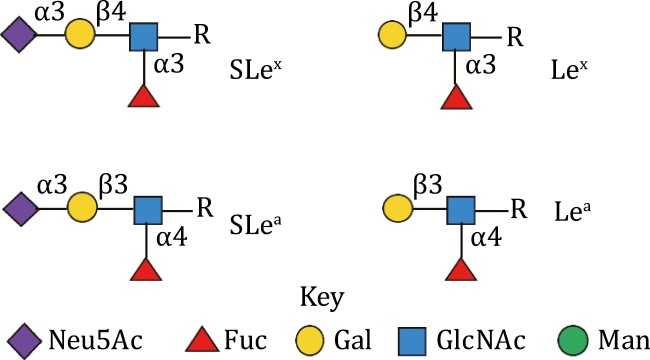
**Structure of siaylated and un-sialylated Lewis** ^**x/a**^ **glycan epitopes**. Sugar residue arrangement and the respective linkages shown to create the SLe^x/a^ and Le^x/a^ structures. These epitopes can be displayed on both N*-* and O-linked sugars, lipid or protein at the “R” position. The minimal glycan epitope for SLe structure is a sialic acid residue α2,3-linked to galactose with a fucose α1,3-linked (SLe^x^) or α1,4-linked (SLe^a^) to GlcNAc. Le structures do not have the terminal sialic acid. Neu5Ac, N-acetylneuraminic acid (sialic acid); Fuc, fucose; Gal, galactose; GlcNAc, *N*-acetylglucosamine; Man, mannose. The glycans are drawn in accordance with GlycanBuilder (Damerell et al. 2012).

DC-SIGN is a type-2 membrane protein that forms stable tetramers in the plasma membrane (Feinberg et al., 2005). This oligomerization is mediated via α-helical coiled-coil domains and leads to the clustering of four C-type carbohydrate recognition domains (CRDs), in close proximity, to produce a multivalent glycan-binding surface (Fig. 1). Each CRD possesses a Glu-Pro-Asn (EPN) motif that preferentially binds to mannose-type saccharides. DC-SIGN has also been demonstrated to interact with fucosylated oligosaccharides, such as is found on Lewis-x (Le^x^; Fig. 2) glycan structures, via an extended binding site that makes multiple contacts with multiple saccharide units within a single oligosaccharide structure (Guo et al., 2004; Feinberg et al., 2007). More recently, further analysis of the binding function of DC-SIGN has demonstrated cues for driving cooperativity in complex ligand interactions, giving rise to higher order interactions that in turn broaden the sensory range of this family of lectins (Wawrzinek et al., 2021). Native DC-SIGN tetramers also have N-terminal transmembrane and intracellular domains, the latter of which carries internalization motifs allowing DC-SIGN to serve as an endocytic receptor on dendritic cells (DCs).

At the turn of the 21^st^ century, the identification that DC-SIGN could interact with human immunodeficiency virus (HIV) pioneered a new way of approaching lectin-pathogen interactions in human disease. Work by Geijtenbeek et al. uncovered how HIV-1 could bind to DCs in peripheral tissues via the viral carbohydrates that are densely presented on the gp120 glycoprotein of the enveloped virus outer surface (Geijtenbeek et al., 2000b). In this scenario, the principal host binding partner for HIV-1 is DC-SIGN (rather than the well-characterized HIV-gp120 host counterpart, CD4). gp120 has around 40% carbohydrate by mass with 23 N-linked glycosylation sites and DC-SIGN binds to high mannose N-linked oligosaccharides presented on this glycoprotein (Feinberg et al., 2001; Mitchell et al., 2001).

Geijtenbeek et al. went on to outline the role of the DC-SIGN-gp120 interaction in the primary capture of infectious HIV particles in areas such as genital mucosa/lamina propria via DCs that are typically resident there (and where CD4^+^ T cells are typically scarce) (Geijtenbeek et al., 2000b). Subsequently, in keeping with normal DC function, these cells would migrate to regions rich in CD4^+^ T cells, such as lymph nodes, where DC-SIGN-bound HIV-1 particles would then have access to abundant populations of the CD4 co-receptor-positive T cells (especially via the DC-T cell immunological synapse) within which the virus can efficiently proliferate (Cameron et al., 1992). This phenomenon has been termed as infection *in trans* and generated new hypotheses around how a persistent pathogen within the host, such as HIV-1, can achieve efficient primary infection of CD4^+^ T cells and potentially be sequestered and trafficked within the body to evade neutralization and prolong survival. The utilization of DC-SIGN in such an orthogonal set of virus–host interactions has been investigated in other infectious diseases, leading to a broadening of perspectives as to how pathogens can subvert host immune responses via pathogen carbohydrate binding by host lectins, especially CTLs. A range of both lectins and pathogens has been demonstrated to contribute to these systems of orthogonal interactions. Pathogens include *Mycobacterium tuberculosis* (Geijtenbeek et al., 2003), and *Helicobacter pylori* (Bergman et al., 2004) and viruses such as influenza A (Hillaire et al., 2013; Yang et al., 2021), Ebola (Simmons et al., 2003), and the severe acute respiratory syndrome coronavirus (SARS-CoV, including SARS-CoV-2) (Chan et al., 2006; Amraei et al., 2021; Cai et al., 2021). In addition, DC-SIGN also binds to the DENV glycoprotein of Dengue virus, promoting virus interactions with human DCs (Navarro-Sanchez et al., 2003; Liu et al., 2017). CTLs of particular interest in relation to infection *in trans* include DC-SIGN and also DC-SIGN-related protein (DC-SIGNR also known as L-SIGN; CD299) and surfactant protein-A and -D (SP-A and -D; Fig. 1) which will be discussed further below.

DC-SIGN has been demonstrated to be exploited by HIV-1 for survival in the host. HIV-1 contact with DC sequesters and stabilizes viral particles in cycling endosomes during the journey to T cell-rich areas (Kwon et al., 2002). Furthermore, the intracellular signaling properties of DC-SIGN invoked by virus contact influence the immunobiological function of the DCs. Gringhuis and colleagues demonstrated that signaling via DC-SIGN stimulates Raf-1 kinase-associated systems that modulate Toll-like receptor (TLR) responses in cells that co-express these receptors (Gringhuis et al., 2007; Gringhuis et al., 2009). The outcome of this modulation of TLR signaling leads to NF-kappa-B p65 acetylation and polarizes the cell towards anti-inflammatory/immunosuppressive responses, such as promoting IL-10 secretion and suppressing IL-12 production. Consequently, the scale-up of this immunosuppressive polarization broadly downregulates responses that would normally combat viruses such as HIV-1. Therefore, this subversion of immune function via engagement with DC-SIGN favors the survival of an exogenous threat (HIV-1). These remarkable insights relating to DC-SIGN led researchers to investigate other major pathogens and especially highlighted *M*. *tuberculosis* as a microbial species that can interact with DC-SIGN (Geijtenbeek et al., 2003). Mycobacterial binding has been demonstrated to occur via their carbohydrate-rich mannosylated lipoarabinomannan (ManLAM) surfaces, supporting internalization into target cells and also immunosuppression with overlapping mechanisms utilized by HIV-1. Furthermore, additional glycoprotein ligands for DC-SIGN have been identified from *M*. *bovis* bacillus Calmette-Guérin (BCG) extracts (Carroll et al., 2010), in particular the lipoarabinomannan carrier protein lprG which can also engage TLR2, and thus suggests that certain pathogens can possess networks of molecules that help to reprogram or polarize the host responses towards an immunosuppressed state that is a hallmark of tuberculosis (van Kooyk et al., 2003).

Beyond DC-SIGN, other CTLs have been implicated in promoting infection and/or pathogen survival within the host, including further exploitation of these lectins by HIV-1. It has been demonstrated that the soluble collectin molecules SP-A and SP-D (Fig. 1) can also interact with the heavily glycosylated HIV particles through their CRDs (which are broadly homologous to the CRD of DC-SIGN). In addition to their presence in pulmonary surfactant, SP-A and SP-D are also present in genital mucosae where HIV-1 can make primary contact with the host. Studies by Gaiha et al. and Madsen et al. have demonstrated that SP-A and SP-D display a dual modulatory role in HIV-1 infection, whereby viral particles can be targets for binding but these opsonized viral particles then become engulfed by DCs and trafficked to the lymph nodes which (as described above) promotes infection (Gaiha et al., 2008; Madsen et al., 2013). DC-SIGNR has also been implicated in HIV-1 infection *in trans*, although its distribution is significantly different from DC-SIGN, being expressed principally in lymph node and hepatic endothelium rather than DCs and selected subpopulations of macrophage (Soilleux et al., 2000; Pohlmann et al., 2001; Soilleux et al., 2002).

As with many immune receptors and interactions, the response can be context dependent, and despite the unraveling of the important roles for DC-SIGN in infectious disease, this has also uncovered a role for the receptor in immune tolerance. Before receiving its moniker as DC-SIGN in 2000, the molecule was previously identified in the placenta (Curtis et al., 1992), and was later found to be heavily expressed on both placental Hofbauer cells and maternal decidual macrophages (Soilleux et al., 2001). This concentration of DC-SIGN at the feto-maternal interface strongly suggests that it is involved in the immunological tolerance that arises during pregnancy and indeed primate models show that DC-SIGN expression in uterine decidua heralds the onset of pregnancy (Breburda et al., 2006). In human pregnancy, downregulation of DC-SIGN on placental Hofbauer cells has been demonstrated to correlate with complications such as pre-eclampsia (Yang et al., 2017). Beyond tolerance in pregnancy, DC-SIGN may also be involved in establishing and maintaining accommodation of allografts, with considerable potential in understanding transplantation immunology (Conde et al., 2015). With further involvement of DC-SIGN in apoptotic cell uptake, consolidation of the immunological synapse via ICAM-3 binding (Geijtenbeek et al., 2000c), support of DC vascular trafficking via ICAM-2 binding (Geijtenbeek et al., 2000a), and engagement of neutrophils by DCs (van Gisbergen et al., 2005), it is evident that the natural function of DC-SIGN in particular lies in moderating the tolerogenic axis of host immunity and inflammation. It is therefore unsurprising that certain pathogens have evolved ligands and mechanisms to exploit such networks to evade eradication by host defenses.

## MBL and the detection of self and non-self glycans

MBL is a member of the collectin family which is synthesized in the liver and circulates in the blood stream. Serum concentrations of MBL in the blood vary greatly between individuals with a mean of 2.4 ± 2.7 µg/mL and is undetectable in the serum of up to 20% of individuals (Mayilyan et al., 2006). This variation is a result of polymorphisms in the coding sequence and/or the promoter regions of the gene for MBL (Madsen et al., 1998). Polymorphisms within the coding region often interfere with the oligomerization of the MBL subunits at the protein level which compromises their avidity of binding sugar-ligands (Larsen et al., 2004). MBL is structurally similar to that of the complement protein C1q (Reid, 2018), composed of monomers of 25 kDa (each with its own CRD) which trimerize via a collagen triple helix, and 2–6 of these timers then oligomerize to form the higher-order structures of MBL that resemble a “bunch of tulips” arrangement (Fig. 1) (Arnold et al., 2005a; Auriti et al. 2017). Although the affinity of a single CRD to a sugar is weak (Iobst and Drickamer, 1994), MBL’s higher-order oligomers facilitate greater overall avidity of binding through permitting engagement of multiple CRDs (Kjaer et al., 2013).

**Figure 3.**
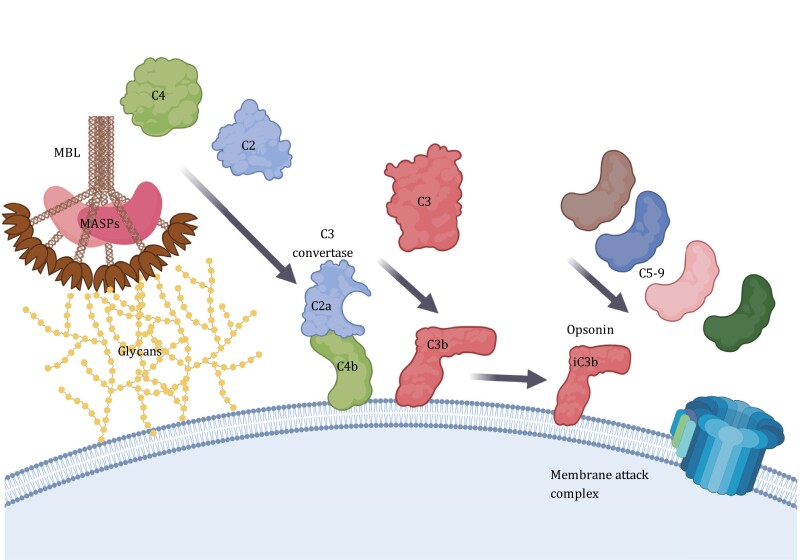
**Lectin pathway of complement activation**. MBL binding to cellular glycans results in the autoactivation of MASP-2 which cleaves C4 to expose a thioester group (not shown but detailed chemistry is presented by Dodds et al. (1996) which allows fragment C4b to covalently bind to proteins and cell membranes in the local vicinity, facilitating its accumulation on the cell surface. C2 subsequently binds C4b and is subsequently cleaved by MASP-2 to form the C3 convertase C4b2a. The C3 convertase then cleaves C3 which exposes its hidden thioester group (not shown) to allow the fragment C3b to accumulate on the cell surface. C3b is further cleaved to form iC3b which is a potent opsonin. Activation of C3 also leads to the formation of the membrane attack complex through the formation of a C5 convertase consisting of C4b2a3b which cleaves C5 which binds to the cell surface and forms a scaffold which associates with C6-9 for the formation of the membrane attack complex which consists of a pore of polymerized C9 which leads to cell lysis. MBL, mannose-binding lectin; MASPs, MBL-associated serine proteases. Created using BioRender.com.

MBL is one of the key activators of the lectin pathway of complement activation through its MBL-associated serine proteases (MASP-1–3) (Presanis et al., 2004; Tsakanova et al., 2018) (Fig. 3). MASP-2 has been demonstrated to be the crucial MASP and is sufficient to activate the complement cascade alone (Thiel et al., 1997; Merle et al., 2015). Upon MBL binding via its CRDs, MASP-2 autoactivates and cleaves complement factor C4 to generate C4a and C4b fragments, which exposes a thioester group which allows C4b to covalently bind to the activating surface (Ambrus et al., 2003; Moller-Kristensen et al., 2007; Schwaeble et al., 2011) (Fig. 3). Subsequently, complement factor C2 then binds C4b to be cleaved by MASP-2 to generate C2a which remains bound to C4b and becomes the C3 convertase (C4b2a). The C3 convertase subsequently cleaves complement component C3 to generate C3b, which covalently binds to proximal surfaces via a thioester group and is subsequently cleaved to form iC3b that can act as a potent opsonin (Fig. 3). Additionally, activation of C3 leads to the formation of the membrane attack complex (MAC) that causes cell lysis (Fig. 3). There is evidence that MASP-2 can directly cleave C3 at a low level (Ambrus et al., 2003) which could explain the residual lectin pathway activity reported in the absence of C4 and/or C2 components (Yaseen et al., 2017). However, unlike MASP-2, the role of MASP-1 and -3 is not as clearly defined. There is evidence that MASP-1 may play a role in activating MASP-2 (Heja et al., 2012) and augmenting MASP-2 activity through directly cleaving C2 (Chen and Wallis, 2004), however, as MASP-1 cannot cleave C4 it is insufficient to fully activate complement on its own. Interestingly, Sim, R.B. and colleagues demonstrated that MASP-1 was capable of cleaving factor XIII and fibrinogen (Krarup et al., 2008) suggesting an alternative role for the enzyme potentially associated with coagulation (Presanis et al., 2004; Krarup et al., 2007). In addition to MASP-1–3, there are also two non-enzymatic MASP splice variants; MAp44 (Degn et al., 2009) and MAp19 (Stover et al., 1999; Iwaki et al., 2006; Degn et al., 2011). Although the role of these splice variants remain debated, there is evidence that they may play a regulatory role for MASP activation (Iwaki et al., 2006; Degn et al., 2009; Degn et al., 2013).

In addition to complement activation, MBL binding can have a direct neutralizing/opsonising role of pathogenic material, where it has been demonstrated to bind to glycans presented on a range of pathogens including; bacteria (Neth et al., 2000), virus (Kase et al., 1999; Ji et al., 2005; Brown et al., 2010; Murugaiah et al., 2021), yeast (Lillegard et al., 2006; Brouwer et al., 2008), and parasites (Green et al., 1994). MBL-bound pathogens are then phagocytosed by macrophages/DCs through MBL receptors to promote clearance (Malhotra et al., 1990; Ogden et al., 2001; Presanis et al., 2003). MBL binding can also have a neutralization effect and can prevent viral entry into cells (Kase et al., 1999; Ji et al., 2005; Brown et al., 2010). The importance of MBL’s interactions with pathogens has been most clearly observed and defined in infants, where low levels of serum MBL have been linked to severe and recurrent infection (Summerfield et al., 1997; Faber et al., 2007) and greater risk of developing neonatal sepsis (Gao et al., 2015). In adults, low levels of circulating MBL have been associated with recalcitrant rhinosinusitis (Justice et al., 2015) and increased mortality from *pneumococcal* infection (Eisen et al., 2008). However, other studies have failed to find such links to suseptability to *meningococcal* disease (Bradley et al., 2012). Despite these important roles for MBL in controlling infection, these pathways have also been exploited by some pathogens to improve infection. In another example of “friend” turns “foe”, it has been demonstrated that intracellular pathogens like *Leishmania*, exploit the opsonizing capabilities of MBL to actively facilitate their entry into cells (Santos et al., 2001). Interestingly, MBL can also play an immune-modulating role when bound to pathogenic material. For example, MBL-opsonized material has been shown to influence the functionality of monocyte/macrophages to TLR ligands, reducing their expression of pro-inflammatory cytokines such as IL-1α and IL-1β and increasing their expression of the anti-inflammatory cytokine IL-10 upon lipopolysaccharide (LPS) stimulation (Fraser et al., 2006). This effect is not just restricted to myeloid cells but also lymphocytes where T cell engagement with MBL’s collagen-like domains by calreticulin on the T cell surface reduces proliferation and T cell receptor signaling (Zhao et al., 2017). These modulating roles of MBL, which skew a more anti-inflammatory state of the host immune response, potentially act as a mechanism to facilitate the resolution phase of the immune reaction. MBL’s role in modulating the immune response could also help to explain the link between low serum MBL levels and auto-immune disease (Garred et al., 2000; Tsutsumi et al., 2005).

**Figure 4.**
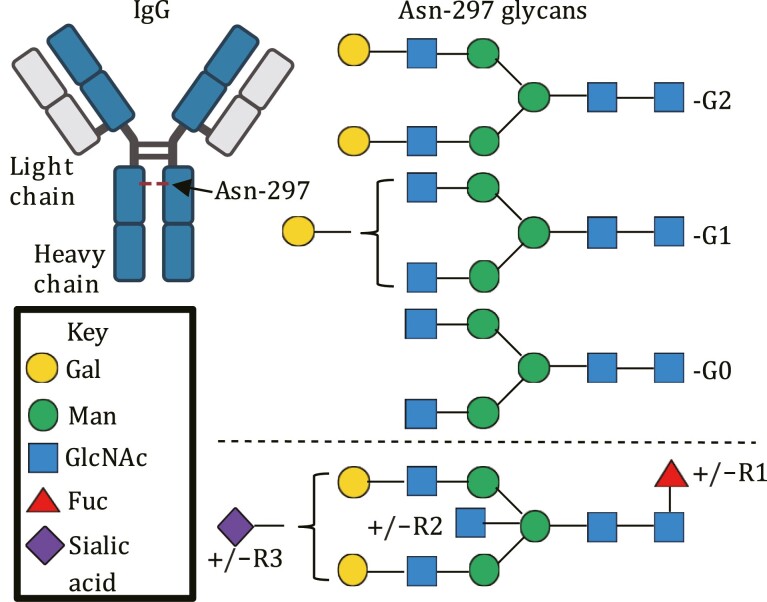
**The glycoforms of IgG**. Diagram (left) shows the structure of IgG highlighting the approximate location of the heavy chain N-linked glycosylation site at Asn-297 in the Fc region (red dash). The predominant glycan structures that occupy the Asn-297 site on each heavy chain are displayed (right top) and form the basis of the IgG-G0, -G1, -G2 nomenclature based on the number of terminal galactose residues. The glycans shown may also vary by the presence of absence of a core fucose (R1) and/or bisecting GlcNAc (R2) and/or sialic acid (R3) (right bottom). Asn, Asparagine; Fuc, fucose; Gal, galactose; GlcNAc, *N*-acetylglucosamine; IgG, immunoglobulin G. The glycans are drawn in accordance with GlycanBuilder (Damerell et al. 2012). IgG was created using BioRender.com.

Up to the mid-1990s the role of MBL was exclusively associated with detecting and clearing pathogens, however, a seminal article from Sim, R.B. and colleagues changed this perception of CTLs when they demonstrated that MBL could bind to a glycoform of the host protein immunoglobulin G (IgG) and activate complement (Malhotra et al., 1995). IgG has a single conserved N-linked glycosylation site at asparagine (Asn)-297 on each of its heavy chains which are situated in the Fc portion of the molecule (Fig. 4). The glycans which occupy this site are generally highly processed to terminal galactose and sialic acids (Arnold et al., 2005a), however in the serum from healthy individuals about 25% of IgG glycans are missing these sugars and display terminal GlcNAc residues, referred to as “IgG-G0” glycoforms (for their lack of terminal galactose; Fig. 4) (Parekh et al., 1985; Arnold et al., 2007). Interestingly, in chronic inflammatory conditions such as rheumatoid arthritis (RA) and osteoarthritis, the prevalence of IgG-G0 significantly increases to >50% of the IgG pool (Parekh et al., 1985). Subsequent studies identified increased IgG-G0 in a range of inflammatory conditions including Crohn’s disease, juvenile onset chronic arthritis, systemic lupus erythematosus complicated by Sjögren’s syndrome and tuberculosis (Parekh et al., 1985; Parekh et al., 1989; Bond et al., 1997). Serum elevations of IgG-G0 in chronic inflammatory conditions appear to be directly linked to the underlying inflammatory state, as IgG-G0 levels in patients with RA return to baseline levels during pregnancy when there is a remission of the disease (Rook et al., 1991). Other CTLs and CTLRs have been demonstrated to interact with IgG-G0 glycans such as macrophage mannose receptor (MMR) (Dong et al., 1999) (Fig. 1). Although Fc receptor-mediated interactions have been demonstrated to play a major role in IgG-G0 cellular effects (Nimmerjahn et al., 2007), the observation by Sim et al., (Malhotra et al., 1995) provided added functionality to MBL and identified that the crosstalk between the complement system and IgG was not just through C1q and the classical pathway of complement activation, but also extended to the lectin pathway (Fig. 3).

MBL’s interaction with host glycoproteins is not just restricted to IgG. MBL has also been demonstrated to bind glycoforms of human IgM which are enriched for their presentation of terminal GlcNAc residues (Arnold et al., 2005b) and dimeric/polymeric forms of IgA (Roos et al., 2001; Royle et al., 2003). Furthermore, MBL interacts with oligomannose glycans present on the serum thioester protein α2-macroglobulin (Arnold et al., 2006b). MBL has also been demonstrated to interact with populations of host cells including B-cells (Downing et al., 2005), DCs (Downing et al., 2003; Downing et al., 2005), senescent fibroblasts (Tomaiuolo et al., 2012) and apoptotic cells (Ogden et al., 2001) through its CRDs in a sugar-dependent manner. Clearance of apoptotic cells has emerged as a significant function of MBL which has been demonstrated elegantly *in vitro* and *in vivo* (Stuart et al., 2005). These studies highlight the diverse roles of MBL, and CTL interactions more generally, which can play pivotal roles in both pathogen identification but also provide a mechanism of cross-communication between host proteins associated with the immune response.

## Selectins and cell trafficking

**Figure 5.**
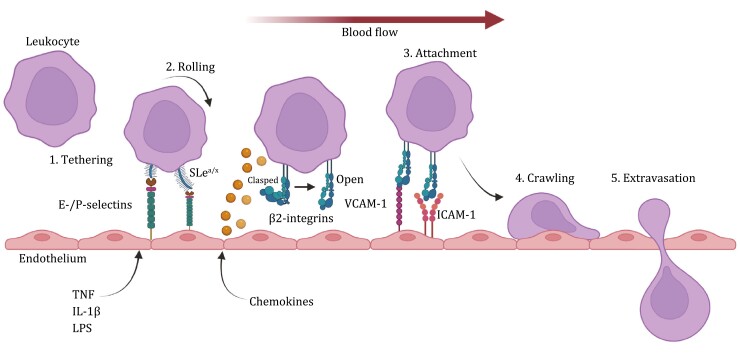
**The steps of leukocyte extravasation from the blood**. Diagram showing the key steps and interactions associated with leukocyte extravasation (from left to right). Inflammatory stimuli from the tissue such as TNF, IL-1β, and LPS activate the endothelium to express high levels of E- and P-selectin which act to tether/capture leukocytes from the blood via SLe^x/a^ epitopes presented on leukocyte glycoproteins. This initiates leukocyte rolling via these CTL interactions. Leukocytes then become activated in response to chemokines and other chemoattractants being released from the inflammatory site which activates β2-integrins that adopt a structural change that allows more efficient interactions with endothelial VCAM-1 and ICAM-1 permitting firm attachment and arrest. Subsequently, this leads to a slow crawling of the leukocyte prior to transendothelial migration into the tissue. ICAM-1, intercellular adhesion molecule-1; IL-1β, interleukin-1β; LPS, lipopolysaccharide; TNFα, tumor necrosis factor-α, VCAM-1, vascular cell adhesion molecule-1. Created using BioRender.com.

Selectins are a type I membrane class of CTLR which bind sialoglycans. There are three members of the family; L-, E-, and P-selectin, named after their expression on leukocytes, endothelium, and platelets, respectively (Fig. 1). However, their expression is not as selective as their name infers where, for example, endothelial cells express both E- and P-selectin (McEver, 2015). Selectins have specificity for sialyl Lewis-x (SLe^x^) and its isomer glycan, sialyl Lewis-a (SLe^a^) (Fig. 2). The minimal glycan epitope for SLe^x^ is a sialic acid residue α2,3-linked to galactose with a fucose α1,3-linked to GlcNAc. The interaction between SLe^x/a^ epitopes and E- and P-selectin expressed by endothelial cells has emerged as a crucial interaction in facilitating leukocyte recruitment to inflammatory sites, which has been extensively reviewed (McEver, 2015). Although there are sites where E-selectin is constitutively expressed, such as the bone marrow (Schweitzer et al., 1996), it is not present on most endothelial cell surfaces during homeostatic conditions, but is highly inducible in the presence of inflammatory stimulants such as tumor necrosis factor-alpha (TNF), IL-1β and LPS (Wong and Dorovini-Zis, 1996). Leukocyte engagement with selectins expressed on endothelial cells via SLe^x/a^ epitopes presented on their surface provides the initial anchoring interaction which permits slowing and rolling of leukocytes along the endothelium (Kunkel and Ley, 1996) (Fig. 5). Once slowed against the sheer force of the blood stream, leukocytes become activated by chemokines and other chemoattractants that are diffusing from the inflammatory site (or expressed by the activated endothelium) which results in the activation and engagement of β2-integrin interactions on the leukocyte surface (Alon and Feigelson, 2012; Lefort and Ley, 2012) (Fig. 5). In the resting state, β2-integrins adopt a “clasped” inactivated heterodimer form, but once activated, a structural change permits more efficient integrin interactions (Alon and Feigelson, 2012; Lefort and Ley, 2012) (Fig. 5). Leukocyte β2-integrins permits a firmer adhesion through engagement with intercellular adhesion molecule-1 (ICAM-1) and vascular cell adhesion molecule-1 (VCAM-1) which are expressed on activated endothelium and results in migration arrest and crawling of the leukocyte with eventual transmigration through the endothelial layer into the site of inflammation (Fig. 5). CTL interactions are fundamental to leukocyte recruitment to inflammatory microenvironments. However, such interactions in chronic inflammatory conditions can exacerbate disease pathogenesis, such as is observed in arthritis (Hopkin et al., 2019) and cancer (Natoni et al., 2016). The implications of selectins in cancer will be discussed in the next section.

## CTLs and tumor progression

Changes in glycosylation patterns in cancer are common and can be associated with genetic alterations (Radcliffe et al., 2007) or cancer-associated inflammation which modifies cellular glycosylation processing machinery (Arnold et al., 2008; Pinho and Reis, 2015). Two if the most well-characterized N-linked glycosylation changes in cancer are associated with the degree of branching, dictated by the number of GlcNAcs attached to the chitobiose core and the prevalence of SLe^x^ (Saldova et al., 2007; Arnold et al., 2011) (Fig. 2). As described in the previous section, selectins bind SLe^x^ glycan epitopes on leukocytes to promote their recruitment from the blood, which can be exploited in cancer to recruit pro-tumoral cell types such as macrophages into the tumor which facilitate disease progression (Muliaditan et al., 2018; Opzoomer et al., 2021) and metastasis (Laubli and Borsig, 2010; Natoni et al., 2016; Esposito et al., 2019). SLe^a^ epitopes (Fig. 2) on the surface of tumor cells promote their rolling and extravasation from the blood stream (Ben-David et al., 2008; Heidemann et al., 2014) and facilitate metastatic niche formation (Laubli et al., 2009). In acute myeloid leukemia (AML), E-selectin helps to retain tumor stem cells within a bone marrow vascular niche which protects these cells from the effect of cytotoxic chemotherapeutics (Barbier et al., 2020).

Follicular lymphoma (FL) is a B cell malignancy that represents about 40% of all non*-*Hodgkin lymphomas. A seminal article by Stevenson et al. demonstrated that aberrant N-linked glycosylation sites in the B cell receptor (BCR) Fab region of the surface immunoglobulin (Ig) were common, almost a characteristic feature, in FL (Radcliffe et al., 2007). These N-linked glycosylation sites were introduced though opportunistic selection during somatic hypermutation in the Ig Fab variable region of the BCR. Despite the apparent accessibility of the N-linked glycans for processing to higher order sugars, the glycans attached to the Fab in FL remained relatively unprocessed high oligomannose glycans which were targets for CTL engagement (Radcliffe et al., 2007). Since this seminal article, aberrant high oligomannose glycans on the surface Ig in FL have been demonstrated to interact with CTLRs such as DC-SIGN (Coelho et al., 2010; Amin et al., 2015; Linley et al., 2015; Valle-Argos et al., 2021) and MMR (Coelho et al., 2010) (Fig. 1) which provide an antigen-independent activating signal to the B cells. Macrophages which reside in the tumor, referred to as tumor associated macrophages (TAMs), are a highly plastic stromal cell type which facilitate cancer progression (DeNardo et al., 2009; Murray et al., 2014; Mantovani et al., 2017; Muliaditan et al., 2018; Opzoomer et al., 2021) and express DC-SIGN (Amin et al., 2015) and MMR within the tumor microenvironment. More recently, it has been demonstrated that CTLR engagement with the surface Ig Fab glycans in FL acts to block higher-affinity antigen engagement as a mechanism to protect the B cells from over-stimulation while concurrently providing a persistent low-level activation signal to these cells (Valle-Argos et al., 2021), providing a driver mechanism for cancer progression that is driven by CTL binding.

Tumor cells can present aberrant glycosylation which permit binding of CTLs, such as MBL (Terada et al., 2005). DC-SIGN binds Mac-2-binding proteins (Mac-2BP) expressed by a variety of colorectal cancer cell lines through their α1-3/4-fucose moieties of Le glycans, where engagement attenuates DC maturation (Nonaka et al., 2011). DC-SIGN can also engage with Le glycans on carcinoembryonic antigen (CEA) and CEA-related adhesion molecule-1 (CEACAM1) (Nonaka et al., 2008). *In vitro* studies have demonstrated that this interaction also modulates DC maturation and skews their cytokine profiles to a more immune-modulatory phenotype, secreting higher levels of IL-6 and IL-10 (Nonaka et al., 2008). As such, the engagement of DC-SIGN to aberrant cancer glycans has the potential to contribute towards suppressing the anti-tumor immune response. This is supported by the observation that conditioned media from tumor cell: DCs co-cultures was able to suppress Th1 responses *in vitro* (Nonaka et al., 2008). Another CTL, Mincle (Clec4e) has also been associated with suppression of T cell mediated tumor control (Seifert et al., 2016). Mincle signaling on TAMs in murine LL2 and B16 tumors has been demonstrated to promote macrophage polarization to a pro-tumoral phenotype which facilitates progression of the disease (Li et al., 2020). The Tn glycan epitope presented on tumor-associated glycoproteins has also been demonstrated to be a target for macrophage galactose-type lectin receptor (MGL, CD301) which can discriminate between healthy and tumor-associated glycans of the mucin MUC1 (Saeland et al., 2007). MGL engagement with the Tn glycans presented on CD45 of activated T cells has also been demonstrated to compromise T cell proliferation and activity (van Vliet et al., 2006). However, not all CTLR engagement in cancer results in a pro-tumoral function. For example, Dectin-1 expressed on macrophages and DCs has conversely been demonstrated to play an immune-licencing role for natural killer (NK) cell activation to facilitate an anti-tumor immune response (Chiba et al., 2014). Dectin-1 signals through an interferon regulatory factor-5 (IRF5)-dependent pathway (del Fresno et al., 2013), which provides a pro-inflammatory activating signal for DCs. Dectin-1 engagement with aberrant N-linked glycosylation presented on murine B16 melanoma cells improved DC’s licencing of NK cell killing function which prevented tumor formation (del Fresno et al., 2013). As such, in chronic inflammatory conditions CTL and CTLR interactions can exacerbate the underling disease pathology and be exploited by cancer to facilitate disease progression, where such axes have started to be explored as therapeutic targets.

## Therapeutic opportunities and conclusions

**Figure 6.**
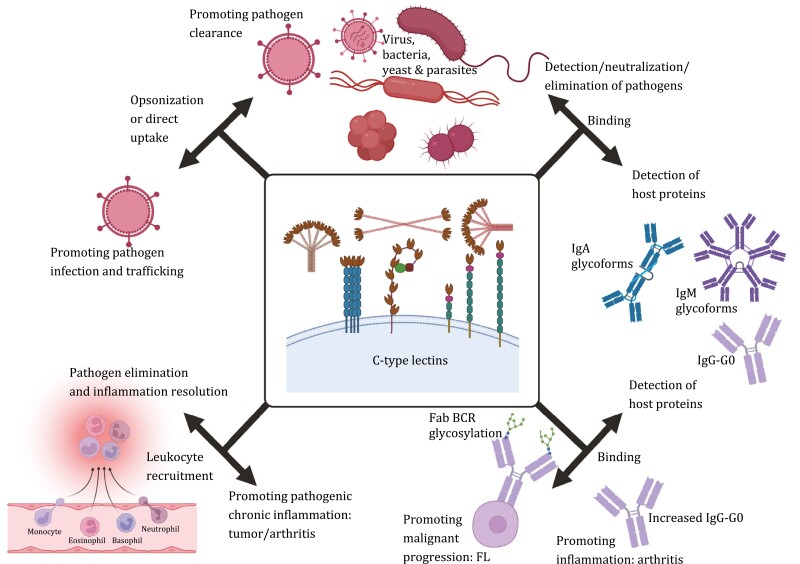
**The diverse roles of CTLs in the defense and promotion of disease**. Diagram summarizing the key axes discussed in the review depicting the role of CTLs and their ‘double agent’ roles in both pathogen and host glycoprotein interactions and the defense and promotion of pathogen, inflammation and inflammatory disease. Centre image is taken from Fig. 1. BCR, B cell receptor; Fab, Ig antigen-binding region; FL, follicular lymphoma; IgG/A/M, immunoglobulin-G/A/M. Created using BioRender.com.

Following the emergence of CTLs onto the scientific stage around forty years ago (Ashwell and Harford, 1982), the understanding of their function and value has grown from initial impressions of glycoprotein clearance and microbial recognition to broad and deep participation in mammalian homeostasis, immunity and host-pathogen relationships, inflammation control, and tumor survival mechanisms (Fig. 6). This increase in valuable knowledge has been accompanied by the rapid expansion of glycomics as a major scientific discipline in the 21^st^ century (Guile et al., 1996; Mrksich, 2004). Significantly, the impact of greater knowledge of CTLs has recently led to promising new therapeutic opportunities.

As described in this review, CTLs play an important role in both the detection of pathogen- and host-associated glycans and these axes have considerable opportunity for therapeutic intervention and modulation (Fig. 6). CTLs can be used as recombinant therapeutic preparations or be targets of carbohydrate-conjugated pharmacological agents. E-selectin contributes to chemotherapy resistance in AML has been targeted therapeutically in patients using the glyco-mimetic drug, GMI-1271, which improves responses to chemotherapy (Garcia-Manero et al., 2017) and is currently in a Phase III trial for relapsed and refractory AML (NCT03616470). Chimeric antigen receptor (CAR) T cell immunotherapy, which involves the genetic modification of a patient’s T cells to express activatory CARs with specificity for tumor associated antigens for eventual reinfusion back into a patient (Kosti et al., 2018), have been glyco-engineered to present increased SLe^x^ epitopes to facilitate their accumulation in the solid tumor microenvironment (Mondal et al., 2019). There are many therapeutic antibodies used in the treatment of cancer (Zahavi and Weiner, 2020) and, interestingly, IgG-G0 is often their predominant glycoform (Mimura et al., 2018). In light of the IgG-G0 glycan interaction with CTLs such as MBL which have the potential to activate complement (Malhotra et al., 1995), it is interesting to consider the potential role of CTLs in the therapeutic response to these antibodies where glyco-engineering approaches can be employed to generate the optimal glycan profiles (Mimura et al., 2018). There has also been considerable interest in utilizing recombinant human SP-D for the treatment of lung diseases, especially in children, to bind pathogens and allergens in the lung via its CRDs, to neutralize the material whilst engaging with immune cells to enhance their anti-inflammatory effects (Strong et al., 2003; Clark, 2010; Arroyo and Kingma, 2021). Within the field of short interfering RNA (siRNA) therapeutics, significant progress has also been made by conjugating specific siRNA oligonucleotides with N-acetylgalactosamine (GalNAc) sugars to engage the asialoglycoprotein receptor (ASGPR; also known as the Ashwell receptor), which is a CTL expressed almost exclusively on hepatocytes of the liver. The ASGPR has unique and very high selectivity for GalNAc and is highly endocytic upon engagement, making it an ideal molecule to target in order to promote exclusive uptake and internalization of siRNA molecules within the liver to correct metabolic disorders (Nair et al., 2014). Recently, the first licensed GalNAc-conjugated siRNA drug, Inclisiran, has been approved for the treatment of dyslipidemia (an abnormal level of lipids in the blood) via knockdown of the *PSCK9* gene in hepatocytes to lower systemic low-density lipoproteins (Lamb, 2021; Santulli et al., 2021). This is expected to be the first of many GalNAc-siRNA therapeutics that will rely on the specificity and function of ASPGR to deliver therapeutics to the liver.

In summary, as discussed here, CTLs and CTLRs play a diverse range of functions which can both promote the clearance of infection and resolution of inflammation but in certain contexts be exploited to aid infection by pathogens and exacerbate inflammatory disease. As our understanding of these axes has become deeper, the knowledge for how and where to therapeutically target these interactions most effectively is equally becoming clearer.

## Abbreviations

AML: acute myeloid leukemia
ASGPR: asialoglycoprotein receptor
Asn: asparagine
BCG: *Mycobacterium bovis* bacillus Calmette-Guérin
BCR: B-cell receptor
CEA: carcinoembryonic antigen
CEACAM1: CEA-related adhesion molecule-1
CRDs: carbohydrate recognition domains
CAR: chimeric antigen receptor
CTLs: C-type lectins
CTLD: CTL-domain
CTLR: CTL receptor
DCs: dendritic cells
DC-SIGN: dendritic cell-specific ICAM-3-grabbing nonintegrin
DC-DIGNR: DC-SIGN-related protein
EGF: epidermal growth factor
Fab: fragment antigen binding
Fc: fragment crystallizable
FL: follicular lymphoma
Fuc: fucose
Gal: galactose
GalNAc: N-acetylgalactosamine
GlcNAc: N-acetylglucosamine
HIV: human immunodeficiency virus
ICAM-1: intercellular adhesion molecule-1
Ig: immunoglobulin
IRF5: interferon regulatory factor-5
Le^x^: Lewis-x
LPS: lipopolysaccharide
MAC: membrane attack complex
Mac-2BP: Mac-2 binding protein
Man: mannose
ManLAM: mannosylated lipoarabinomannan
MBL: mannose-binding lectin
MASP-1-3: MBL-associated serine protease-1-3
MGL: macrophage galactose-type lectin receptor
MMR: macrophage mannose receptor
Neu5Ac: N-acetylneuraminic acid
NK: natural killer
RA: rheumatoid arthritis
SCR: short consensus repeat
SLe^x/a^: sialyl Lewis-X/A
siRNA: short interfering RNA
SP-A/D: surfactant protein-A/D
TAM: tumor associated macrophage
TLR: Toll-like receptor
TNF: tumor necrosis factor-alpha
VCAM-1: vascular cell adhesion molecule-1

## Article dedication

J.N.A. and D.A.M. dedicate this article to professor Robert (Bob) Braidwood Sim, our dearly missed mentor who inspired us with his passion for the complement system and encyclopaedic knowledge of the literature which was enriched by his lab’s research contributions over the years. It was our honor to have been trained by Bob and developed as researchers, and as individuals, through his mentorship and kindness. Bob was a truly exceptional mentor, with a tireless dedication to developing those that worked for him, even years after leaving his lab. Bob was a role model to us all.
